# Becoming Aware of Inner Self-Critique and Kinder Toward Self: A Qualitative Study of Experiences of Outcome After a Brief Self-Compassion Intervention for University Level Students

**DOI:** 10.3389/fpsyg.2019.02728

**Published:** 2019-12-06

**Authors:** Per-Einar Binder, Ingrid Dundas, Signe Hjelen Stige, Aslak Hjeltnes, Vivian Woodfin, Christian Moltu

**Affiliations:** ^1^Department of Clinical Psychology, Faculty of Psychology, University of Bergen, Bergen, Norway; ^2^Department of Psychiatry, District General Hospital of Førde, Førde, Norway

**Keywords:** university students, self-compassion, qualitative study, intervention, stress

## Abstract

This qualitative study investigated ways in which student participants in a three-session self-compassion course became more compassionate toward themselves and challenges related to this change. Ninety-four participants completed an online survey and 12 participants were interviewed face-to-face. First, a thematic analysis of the responses from the online survey was conducted, and then sorted by frequency, indicating their representativeness in the written responses. The following themes were identified: (1) being more supportive and friendlier toward self, (2) being more aware of being too hard on oneself, (3) feeling less alone when having painful feelings, (4) having more acceptance of painful feelings, and (5) feeling more stable and peaceful. These five most frequent themes served as a basis for a structured phenomenological analysis in the next analytic stage. They were used as a template for a content analysis of the interview material. Subsequently, a phenomenological analysis was conducted on the interview transcripts covering the five thematic areas.

## Introduction

Student life is associated with a multitude of pressures. Students often alternate between academic ambition and fear of failure. They must often cope with challenges connected to identity development, establish new relationships and find a healthy balance between work and leisure time. In a historical period with increased market-orientation and neoliberal values, perfectionistic demands seem to increase (Curran and Hill, 2019). Existing research indicates that the prevalence of stress is increasing among students in higher education (Robotham and Julian, 2006). Many students have coping strategies and lifestyle habits that put them at risk for low stress tolerance (Bland et al., 2012). Moreover, a growing body of research indicates a high prevalence of mental health problems among students in higher education, and indicates that these problems may be growing both in number and severity (Zivin et al., 2009; Hunt and Eisenberg, 2010; Twenge et al., 2010; Evans et al., 2018). Studies have reported that a sizable minority of students experience light to moderate mental health problems, and that their prevalence is much higher than in a non-student population within the same age group (e.g., Stallman, 2010; Shot-study, 2018).

Self-compassion is described as a healthy way of responding to failure and distress, and refers to the disposition to meet distress with self-directed kindness (self-kindness vs. self-judgment), to understand that one is not alone in experiencing difficulties (common humanity vs. isolation), and to notice distressing feelings without getting lost in them (mindfulness vs. over-identification) (Neff, 2003a). Learning to be compassionate toward oneself might help individuals develop their ability to recognize needs, and motivate themselves toward changes that are meaningful to them. Moreover, a self-compassionate stance may help in coping with challenging emotions with a greater degree of understanding and acceptance. Leary et al. (2007) suggest that self-compassion may buffer people against negative self-directed feelings during distressing social events, and also make them more able to evaluate themselves and their possible shortcomings without being overwhelmed by negative emotions. In a meta-analysis, Zessin et al. (2015) found a positive relationship between self-compassion and psychological well-being, and found that this association was somewhat stronger for cognitive and psychological well-being than for affective well-being.

Given this context, this qualitative study explores the issue of how university and university college students experience self-compassion interventions. Knowledge about students’ lived experience with both challenges and positive outcomes when working with self-compassion in everyday life can offer clinicians and researchers valuable hypotheses about effective change processes, which may in turn guide future practice. To date, only a few studies have examined the effect of self-compassion interventions for university students. Among them are studies that suggest that a mindful self-compassion (MSC) course can increase self-efficacy and optimism (Smeets et al., 2014) and reduce negative thoughts and emotions (Arimitsu, 2016).

This study is concerned with possible effects of trying to help students to become more self-compassionate, using a three session self-compassion intervention. In a multi-method project, including a quantitative multi-baseline randomized controlled trial (RCT), we reported the effects of a three-session self-compassion course on healthy and unhealthy self-regulation and symptoms of anxiety and depression in university students. In the quantitative study, we found gains in personal growth self-efficacy and healthy impulse-control and reductions in self-judgment and habitual negative self-directed thinking, as well as increases in self-compassion and reductions in anxiety and depression. Changes remained at 6-month follow-up for personal growth self-efficacy, self- judgment and habitual negative self-directed thinking, as well as for self-compassion, anxiety, and depression (Dundas et al., 2017).

Very few studies have explored how young adults experience the process of establishing a more compassionate way of treating themselves in difficult situations. In a mixed methods study of young women athletes, Ferguson et al. (2014) found that self-compassion was experienced as useful in difficult sport-specific situations, both strengthening perseverance and decreasing rumination. Participants experienced that motivating themselves in a friendly way improved their performance more than aggressive forms of self-criticism. In a phenomenological study of adolescents who had gone through a variety of life difficulties, participants indicated that self-compassion helped them to maintain a positive outlook, self-acceptance, and emotional balance (Klingle and Van Vliet, 2017).

The aim of this qualitative part of our multi-methods project was to investigate the lived experiences of the students who participated in the brief group-based self-compassion intervention (Dundas et al., 2017). Here, we explore the following research questions: what do the students describe, in their own words, as the most important outcome after participating in a brief group-based self-compassion intervention? In what ways did the students become more compassionate toward themselves? What was it about the intervention that contributed to such an outcome?

## Materials and Methods

### Setting

The findings reported in this qualitative study were collected as part of the larger multi methods clinical study conducted on-campus at University of Bergen (Dundas et al., 2017).

### Participants

Initially, 158 participants (85% women, mean age 25 years, standard deviation [SD] = 4.9) were recruited to a study on ‘self-compassion for students’ via information on student websites hosted by a local university and two university colleges during spring 2016. A clear majority of participants were Caucasian and of Norwegian nationality.

Ninety-seven students completed the intervention in one of four identical courses. A *t*-test comparing students who completed vs. dropped out showed that they were similar with respect to gender, age, and baseline values of the variables of interest (Dundas et al., 2017). The 97 completers (80 women, 17 men) represent the full participant sample contributing to the initial qualitative survey in the project. Twelve of these participants contributed to individual interviews in addition to the qualitative survey responses. The 12 participants contributing to in-depth interviews were one man and 11 women with a mean age of 23.

### Intervention

The intervention was based on components of the MSC course developed by Neff and Germer (2013), and elements of Compassionate Mind Training (CMT, Gilbert and Procter, 2006), as well as Mindfulness-Based Stress Reduction (MBSR, Santorelli and Kabat-Zinn, 2009). Three 90-min sessions were delivered over a period of 2 weeks. Sessions were led by the first author; the second author was available in the room during the course to help if any participant needed one-on-one attention.

In the first session, the participants were introduced to mindfulness and self-compassion through a 15- min lecture, followed by short mindfulness and self-compassion exercises, group discussions, and experiential practices. In the experiential practices the task is to immerse themselves in experiences (for instance, to consider how they treat themselves and how they treat others in difficult situations), and then reflect upon these practices. The second session dealt with mindfulness, common physical stress reactions, shame reactions, how to cope with destructive self-criticism, how self-compassionate behavior might influence the body and mind, and activating and soothing affective systems within an evolutionary and attachment based framework (Gilbert and Procter, 2006). The final session comprised discussions on positive feelings, reflections on how one wants to live, and further discussions about compassion for oneself and others, adapted from Germer and Neff (2016).

Participants were provided with a link to an audiofile with guided mindfulness and self-compassion exercises for daily use between sessions, as well as copies of the presentations given in each session. Between the first and second sessions, we encouraged participants to use the 15-min audio guides to practice ‘affectionate breathing’ and ‘loving kindness for ourselves’ on a daily basis. These exercises were adapted from the MSC program (Neff and Germer, 2013). Between the second and third sessions, participants were expected to use the audio guides to practice two new 15-min exercises: ‘mindfulness of breathing, body, and emotions,’ adapted from the MBSR program (Santorelli and Kabat-Zinn, 2009), and ‘giving and receiving compassion,’ adapted from the MSC program (Neff and Germer, 2013).

### Design and Data Collection Method

#### Questionnaire

Shortly after the courses, all participants who completed the four self-compassion courses (*N* = 97) filled out an online questionnaire. They were asked to rate the usefulness of the course on a four-point scale from not at all useful to very useful. Those who rated the course as “a little,” (*n* = 14) “somewhat,” (*n* = 37) or “very” (*n* = 47) useful, in sum, all but three respondents (missing = 2, not at all = 1), were provided with an open-ended follow-up question: what was the most important thing you got out of the course?

#### Interviews

When they signed up for the course the participants were informed that they might be contacted for an interview. Following this procedure for the full completer sample, a sub-sample of 12 of the 97 participants was randomly selected and contacted by phone, and asked to participate in a one-on-one in-depth interview. The interviews were conducted face-to-face, 2 to 4 weeks after the course, in the interviewers’ offices, and lasted 30–45 min. All participants who were invited agreed to be interviewed. Interviews were semi-structured and conducted by the third and fourth author, who had not participated in facilitating the course. The interview guide was not based on any specific theoretical approach, although it was informed by Neff’s (2003b) conceptualization of self-compassion. It aimed at an open-ended phenomenological exploration of the participants experiences. The interview started with an open question about what was the most important thing they got out of the course. If the participants described that they treated themselves in other ways than before the course, or motivated themselves in new ways, they were asked specifically to describe situations in which they did so. The interview also explored whether the participant believed that the course had influenced how they handled exams and evaluations, and possible changes in relationships. The last part of the interview focused on the participant’s experience of the course and its different components, and also on possible negative experiences or negative outcomes. The interview guide is included as Appendix.

### Methodological Approach and Data Analysis

We chose a qualitative method that retains the thematic content of the interviews to be able to trace subtle nuances of client perceptions (Braun and Clarke, 2006). This explorative-reflexive thematic analysis was conducted with hermeneutical-phenomenology as an epistemological premise (Laverty, 2003; Binder et al., 2012). The commitment to, and preparation for, exploring experience on a concrete level is the basis of the phenomenological element in our approach (Van Manen, 2014). In line with reflexive methodology, we used our dialog with the participants’ narratives and descriptions to explore and reflect upon the interpretive lenses our professional and theoretical backgrounds bring in. Our goal was to establish a reflexive stance, and as much as possible become aware of how our preunderstanding and preconceptions might affect the way the interviews were conducted as well as our analysis of the transcribed material (Alvesson and Sköldberg, 2000; Finlay, 2003). In line with usual practice in reflexive methodology, we present information about our professional background in the “researcher”-section, that might be relevant for how the focus of the study formed and for how data is interpreted. The rationale for the focus on the researchers as well as the participants, is that research is regarded as an interactional enterprise in which the findings are products of the interplay between researchers and participants. We aim to make our collaborative work as a team and our reflexive processes transparent in this article to enhance the quality and trustworthiness to our study.

In order to explore both a large qualitative dataset whilst still maintaining the ability to follow up these themes in-depth we chose to pair a content analysis with a thematic analysis of 12 detailed individuals’ accounts. Using a template of themes derived from the large dataset allowed us to identify how representative overarching themes were for the larger group, whilst exploring in-depth the unique lived experiences of these themes in a smaller sample of individuals. By pairing these methods, we could cross-validate the themes, whether they were still salient in the more in-depth accounts and whether more detailed follow-up interviews identified more central identifying themes.

The analysis followed a two-stage process. The first stage was to analyze the written responses to the open-ended question in the electronic survey (“What was the most important thing you got out of the course?”), which was completed by 94 of the 97 participants. All researchers read these 94 short responses (approximately 100 words per participant). By comparing individual accounts, we wanted to identify both patterns of commonalities and differences in what participants experienced as the most important outcome of the course. The first author identified separable content units that represented different experiences of what the most important outcome of the course was. Second, he edited the text in accordance with those codes into coded groups of text. Then he interpreted and summarized the meaning within each of the coded groups of text fragments into conceptions and overall descriptions of 11 themes. All authors then turned back to the overall text to check whether voices and points of view in the students’ responses should be added, and if there were any themes which could be added or developed further. This resulted in some minor reformulations. The five most frequently mentioned themes were chosen for further in-depth analysis.

**FIGURE 1:**
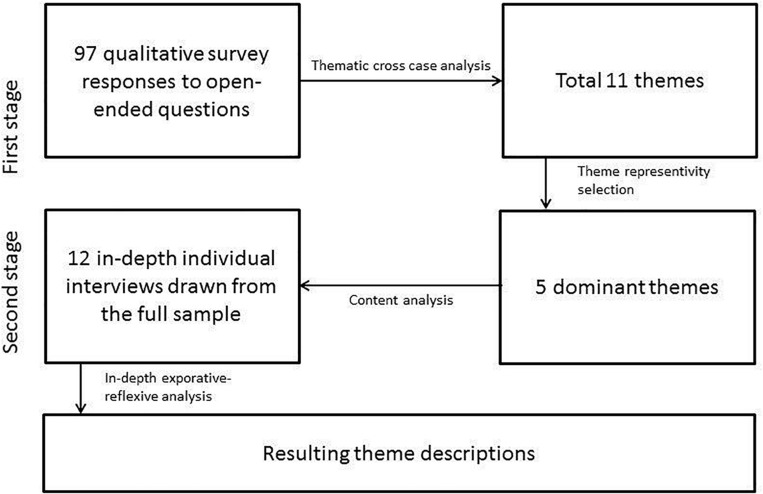
Illustartion of two-stage analytic procedures.

In the second stage of analysis, we analyzed the in-depth interviews of 12 participants by applying an explorative-reflexive thematic approach. See Figure 1 for a visual overview of the analytic procedures.

The analysis of the 12 interviews proceeded through six steps. First, all researchers read all the transcribed material to obtain a basic sense of the participants’ experience. Second, the first author conducted a content analysis, coding the interview-text with the five themes from the stage one analyses as templates. Third, all the other researchers read through the text to check whether they agreed with the first author’s coding, and whether the themes that the templates build upon were representative of the content of the text. After critical examination, the researchers reached consensus on which parts of the text were to be included in the thematic templates. The researcher team agreed that that these templates covered sufficiently large and central parts of the interviews that they could be used for in-depth exploration of the participants experience within the thematic areas. Fourth, the first author interpreted and summarized the meaning within each of the coded groups of text fragments into detailed descriptions of experiences, reflecting those which emerged as the most important aspects of the participants’ experience. Fifth, all authors reread the text to check whether voices and points of view should be added, conceptions and descriptions could be developed further, or correctives made to the preliminary line of interpretation. Sixth, the phenomenological descriptions were finally formulated and agreed upon by all six authors.

### Researchers

The study was conducted within the Research Group for Clinical Psychology at the University of Bergen. The first author is a professor of clinical psychology with 24 years of clinical experience with adults, adolescents, children, and families. His clinical approach is integrative, and he has training in mindfulness- and self-compassion approaches, in addition to Emotion Focused Therapy, and interpersonal/relational psychoanalytic therapy. The second author is an associate professor of clinical psychology with over 30 years of experience working part-time as a clinical psychologist in family and individual settings. Her clinical approach is integrative, and she has training in mindfulness- and self-compassion based approaches, and interpersonal/relational psychoanalytic therapy. The third author is an associate professor in clinical psychology with 11 years of clinical experience with adults, adolescents, and children. She has a special interest in treatment of complex trauma and has training in phase-oriented trauma treatment. Her general clinical orientation is integrative, drawing on humanistic, cognitive, relational, and systemic approaches. The fourth author is an associate professor of clinical psychology with 8 years of clinical experience, and has training in MBSR, Emotion-Focused Therapy, and Cognitive/Systemic Therapy. His general interest is integrative, with an emphasis on humanistic, relational, and existential approaches to psychotherapy. The fifth author is a research fellow and psychologist with 6 years of clinical experience with training in self-compassion and mindfulness-based approaches. The sixth author is a specialist in clinical psychology with 12 years of clinical experience. He holds an adjunct position as professor of clinical psychology, and has training in humanistic and emotion focused approaches to clinical practice. All researchers have previous experience with qualitative research.

### Ethics Statement

The study was approved by the Regional Committee for Medical and Health Research Ethics (Region West) situated at the medical faculty of the University of Bergen, and by the Norwegian Centre for Research Data. Pseudonyms are used for participant quotes in the following to secure their anonymity.

## Results

### First Stage Analysis: Thematic Analysis Across Open Ended Survey Responses

As described as the first stage of the analysis in the methods section, we first conducted a thematic analysis of the 97 written responses to the open ended survey question “What was the most important thing you got out of the course?” We identified 11 themes with varying coverage across participants. Some participants reported one important outcome, whereas others reported several. Table 1 provides an overview of the 11 themes and how broadly they were represented in the material.

**TABLE 1:** Common answers from participants who completed the intervention.

**After the course:**
“I am more supportive and friendly toward myself when things are difficult or feel painful” (*n* = 37)
“I am more aware that I am too hard upon myself or treat myself badly” (*n* = 32)
“I feel less alone in the world when experiencing painful feelings and difficulties” (*n* = 23)
“I am more accepting of painful or uncomfortable feelings, and more easily allow the feelings to be there” (*n* = 22)
“I feel more stable and peaceful, and have learned new ways to cope with stress” (*n* = 19)
“I became more self-reflective” (*n* = 10)
“I become more aware of joyful feelings” (*n* = 10)
“I acquired more knowledge about self-compassion and self-criticism” (*n* = 8)
“I have learned useful tools and techniques that I can use in everyday life” (*n* = 8)
“I became more accepting of my needs” (*n* = 7)
“I am more aware of my feelings and bodily reactions” (*n* = 6)

As can be read from the table, the five most common themes on the open ended survey-question addressed phenomena of supportiveness and friendliness toward self, awareness of harshness toward self, feeling more connected to other people, being more accepting of emotions, and feeling more stable and peaceful in mastering stress. These were carried on as templates for the first step of the analysis of the in-depth interviews. In the following, we present results from the second stage of this analysis in more detail, providing phenomenological nuance through details and quotes.

### Second Stage Analysis: In-Depth Exploration of Identified Themes Through Analysis of Interviews

Original themes from stage one are further analyzed in the second stage, expressing new core meanings arising from in-depth exploration in dialog with the 12 participants.

#### I Am More Supportive and Friendlier Toward Myself When Things Are Difficult or Feel Painful

Participants described having begun a process of treating themselves better in everyday life. They described this as a gradual process of change, where they took one step at a time, often through trying—and sometimes failing—when encountering difficult situations. For example, one participant, Maria, said:

I have really noticed it now during this exam period, that I have been a part of now. With—in a way—not being so hard on myself with thoughts like, ‘I can’t do this.’ But instead trying to turn around and think: ‘Yes, this has been a difficult year.’ And, now—like—I’ve come to think: ‘you’ve done well.’ And try to think a little more positively, and basically be self-compassionate for the situation I’m in.
(…)
S: Sometimes it hasn’t worked so well, but other times it has. In a way, I’ve taken a little breather/‘Think about your breath—let it be in focus’ and thought: ‘You’ve done well.’ Yes. Considering what I have experienced this spring, [I tell myself] ‘it’s okay to be satisfied with what you’ve accomplished.’

This quote indicates that Maria understands her academic struggle in a wider perspective, as an understandable result of difficulties in an important interpersonal relationship this spring. Compassionate breathing is part of the course, and she uses this practice during painful events in her daily life, combining an awareness of the breath with friendly self-talk. Maria experiences herself to still be in the process of moving toward a more accepting and friendly way of treating herself, something she has in common with all the participants that were selected for interviews.

Participants described what they learned as something quite simple, at other times also as something that they were already familiar with. But, paradoxically, they also described it as something new that represented a very important change in the way they treated themselves. On the one hand, being self-compassionate was simple; on the other hand, when it came to actually doing it, it was far from easy. Some participants also highlighted the use of practices and exercises that facilitated a friendly attitude toward oneself. They described it as a form of skill training, and one of them drew parallels to first aid training. The student discussed above, Maria, formulated it this way:

It’s probably the same as with other things, that you practice first aid, right. Then you know that you’re better prepared when a situation occurs. It actually helps to practice being kind toward yourself, so you can use it when it becomes necessary.

#### I Am More Aware That I Am Too Hard on Myself or Treat Myself Badly

Many participants were surprised to find how powerful it felt to become more aware of how harshly they actually treated themselves in difficult situations. They were also surprised at how quickly this awareness made a crucial difference in their lives. The exercise from the first session—how would you treat a friend in a difficult situation, and how would you treat yourself?—was described as a very important experience for many of them. One participant, Inga, described it as a ‘wake-up call’:

We were presented with a situation (….), and then we were supposed to write down how we thought we would react to it, and how we would’ve reacted if a friend were in the same situation. And then I noticed a huge difference in the way I treat myself and how I would treat a friend. In a way, that was the first wake-up call that I had during the course.
I: How did that affect you?
S: It got me to realize that I am really hard on myself, and he talked about the inner critic, and I realized that for me, it’s really mean (…) It got me to really think about, for the rest of the day, to think about all the times I’ve probably been a little hard on myself, but in a way, not really realized it.

In the short run, this insight was a source of worry for her. The night after she experienced this ‘wake-up call’ she felt restless, and repeatedly asked herself why she treated herself so badly. It seemed she balanced on a thin line between rumination and constructive self-reflection. After some days, she landed on the self-reflective side, and made plans for how she could treat herself better in difficult situations. This led her to experience more mastery and positive feelings. It was also described as useful by many participants to externalize and personify their own self-criticism as ‘an inner critic.’ They spontaneously used this term to describe a harsh part of themselves that became active in difficult situations and made them feel more stressed, ashamed or anxious. Inga explained:

I feel like I’ve become more aware, especially of the inner critic they talk about. That I am a little more aware of when it starts to talk, and pay attention when: ‘Oh, no, now I started to think a little negatively.’

The times when she became aware of this, she consciously provided herself with support. Another participant, Karen, described her inner critic in even more personalized terms:

The biggest relief was giving the inner critic a pat and saying, yeah, yeah, now, you can just sit here and complain, I’ll get back to you.
I: So that you, in a way, put it on hold…
S: Yes, I get to put it on hold for a little bit or get a little distance to the constant train of talk, and yes… When I am aware of it, it’s not so intense.

For her, as for several other participants, becoming aware of destructive self-criticism in itself seemed to soften it, something that seemed to create a space for more self-accepting attitudes:

I actually feel like I’m a little more accepting when things go wrong, or things don’t go as well as I had hoped. Then instead of putting myself down, I can, in a way, be a little more accepting, and instead focus on what I can do here and now to improve it.

#### I Feel Less Alone in the World When I Have Painful Feelings and Difficulties

Participants expressed that the way the course highlighted suffering as a natural part of the human condition, as something that everyone had to cope with, albeit in different forms, was experienced as a relief. This topic was both a part of the psychoeducational curriculum of the course and something that was addressed in discussions, inquiry and in the exercises. One participant, Thea, described how insight into the commonality of her difficulties itself made a huge difference:

It was that feeling of: Yes! I’m not alone—in a way. Kind of like, I became a little calmer. And kind of relieved that it’s okay. (…) A reminder that it’s okay that you can’t do it all the time. But it was interesting to experience that when I went through kind of bad things too (…) I could feel that I was in it then.

Thea seemed to feel less lonely and this helped her mobilize resources to deal with difficult experiences. She did not have to fight painful feelings as much; she could more easily accept them. Her experience of being part of a group with ordinary young adults who also faced challenging emotional situations even evoked positive feelings, and made it possible for her to be more present.

Another participant, Elisabeth, explained that she applied the insight that suffering was something she had in common with others as a way to comfort herself. She could remind herself that ‘Yes, that’s human.’ Other participants said that this experience of sharing a common humanity made them feel less lonely, safer, and less ashamed. The recognition that others also face painful feelings and difficult life situations also became evident to participants in more implicit ways, through sharing in the group, structured exercises, and dialogs between participants.

However, some of the participants also felt ambivalent about sharing, and described it as somewhat anxiety-provoking to suddenly have to share experiences from the exercises with the stranger sitting next to them. For instance, Helga stated:

That was probably the part of the course that I feel like didn’t quite work. It was really dependent on who you were talking to and your chemistry. And I feel like, of the people I ended up with, there weren’t that many who were as extroverted. Plus it’s like… you have to let your guard down really fast. So I don’t think I got so much out of that [part]. I didn’t learn that much from pairing into groups of two and talking with my neighbor.

Despite the fact that it was anxiety-provoking for some, many participants described talking with others as a very important and useful part—perhaps even the most important part—of the course. For example, Olga described sharing as a way to discover something about herself:

So it wasn’t just sharing with her, but it felt like I was sharing it with myself for the first time too. But because she responded so well, that nervous feeling went away really fast, and I got a lot out of it. I was left with the feeling of, yes… yes, that was a little scary, but also… I’m so glad I was brave enough to do it. Yes.

The ‘self-compassion pause,’ which is taught during the first part of the course, consists of three parts—recognizing painful emotions, reminding oneself of how suffering is part of the human condition, and then turning to needs—‘what do I need when I feel pain like this.’ The notion that distress is a common human feeling was described as useful and thought provoking. However at least one participant expressed ambivalence regarding being reminded that suffering is part of the human condition. In some social situations the notion that suffering is part of the human condition and that ‘all people have problems’ can be used defensively, for instance by health personnel treating suffering individuals, which can be experienced as a rejection by the patient. Instead of hearing: ‘you’re not alone,’ participants may hear ‘you’re not so special and I don’t care about you.’ One participant had recently experienced a situation like that, and this made the topic problematic to her. For this participant, Andrea, step two of the self-compassion pause became difficult to handle:

S: That step number two, where you acknowledge that others experience the same thing.
I: Yes?
S: There I like got a little, pissed—because that’s exactly what I’ve heard from the doctor and the nurse after the operation.
I: What did they say to you?
S: Well, (…)it was like: —Yes, the operation didn’t go well, but there are a lot of other people who’ve experienced that, there are lots of people who have had to quit the sport—that’s just how it is.

After a while, she managed to differentiate between this very frustrating communication at the hospital and the way the topic of common humanity was addressed in the course. Andrea explained that ‘(I) think it was a little better to hear it during the course [rather than at the hospital] because then it had a [different] context instead of just being an isolated message.’ Step three in the exercise became very important to Andrea, she felt relieved when she turned her attention to her own needs: ‘So I got pretty mad, but then it continued to the third step where it was: what do I need now? And it was actually for me just to continue to see if I can get it [to work in a different way]. And then I felt a little better again.’

#### I Am More Accepting of Painful or Uncomfortable Feelings, and More Easily Allow the Feelings to Just Be There and Not to Escalate

Many participants expressed that participation in the course made it easier for them to accept painful and uncomfortable feelings, and even to provide themselves ‘with some help’ rather than letting difficult feelings escalate, as one participant said. This, in turn, made them feel more safe and secure. They had more faith in their own inner resources and ability to handle difficult emotions, as Anna described:

Like, if I feel uncomfortable for whatever reason, then I think like, I can help myself then in a way. I don’t know if it makes sense, but it’s like I don’t just need to wait for it to pass (…) I think more about what I feel and such. Kind of like what the instructor talked about actually, that you let the feeling be there, but at the same time there’s a kind of security at the other end (…).

I: So there’s a kind of safety, it’s totally okay, to feel that emotion, that it can be there without getting pulled in a sort of downward spiral.
S: Yes. Actually that. I think I’ve avoided a lot of uncomfortable feelings before, because I’m probably prone to thinking negatively. And now I experience them more, but with a kind of security.

In this way, Anna felt able to support herself when difficult emotions awoke. She seemed more able to differentiate between the painful feeling in itself and the cascades of negative thoughts that also arose in these situations. Accepting the feeling and treating herself in a friendly manner seemed to make her more able to decouple from her former thought patterns. In this way, she could step out of rumination before negative thoughts made her feel worse. We also see from this example that she seemed to feel more autonomy when she felt that she had resources to master a difficult situation on her own.

Another participant, Emma, described how she became more aware of what she needed in situations that made her angry, which increased her ability to handle her needs in an autonomous way:

If I get like that, I can get really mad for example, and instead of thinking: Oh, I’m so mad and I’m so dumb, and just becoming angrier and angrier… I think that: Yeah, yeah, it’s not that strange(…) For example, if I get in a fight with my boyfriend, I often want his validation that I am good, even if I am so angry, but he doesn’t want to give me that because he’s really mad at me. Now I’ve started to think, or understood, after that course, that I can give it to myself then. I don’t need him to give it to me, in a way, a validation that I am actually a good person even if I’m mad and act stupid. That there are—like—reasons for that. So that’s really helped. Then, I don’t get as mad either.

Some of the basic assumptions in the curriculum of the course were already familiar to some of the participants, especially when it came to the value of allowing and accepting negative emotions. Some of them were psychology students while others were familiar with the ideas due to the popularization of mindfulness in the media and on the internet. The experiential nature of the course, and the weight given to exercises and practical tools, were described as providing a direct experience that had a distinct impact compared to a more theoretical understanding:

It [the course] was actually a tool for how to practice something I’ve understood purely theoretically. Let feelings be there, as they are, accept them as they are, make space for them, give them room. And I’ve tried that, but not really understood how to do it, so I felt that the methods and techniques we learned were a really good way of practicing what I’ve tried to do.

This participant, Else, said that participation in the course gave her useful tools to deal with difficult situations, and changed the more abstract idea of acceptance into something practical and real that she could use in her own life. Acceptance of painful feelings seemed to lead to an experience of robustness and increased autonomy when handling everyday life challenges. Participants seemed to feel more confident that they could handle challenges, both when it came to academic demands, and in relation to difficulties in their relationships with others.

#### I Feel More Stable and Peaceful, and Have Learned New Ways to Cope With Stress

Stress management was not defined as part of the curriculum of the course *per se*, but several participants described stress-management as the most important outcome. Friendliness toward oneself, especially physically, was often an implicit or explicit part of their new ways of handling stress, as Emma describes when asked about changes after the course:

That I am much better at listening to my body when I should take a break and relax. And then tell myself that that ‘It’s okay, it’s okay to do that.’ Yeah…. Those are the biggest changes.

Emma described a more caring attitude toward herself when it came to basic physical needs, such as the need for rest when she was tired. Following this, she became more accepting toward the emotional body, and gained trust in the ways that emotions can physically be contained and processed:

I choose to like, go into it, (…)yesterday, for example, I got really stressed for a lot of reasons, and in addition, I was really exhausted. And I started to rile myself up and started thinking; ‘Oh my God, it’s going to go wrong and everything is going to go badly,’ and really like worst case scenario thoughts and catastrophizing, and then I started to like, cry and then instead of just laying down and continuing to think and not going out, I laid down on the bed for a little bit and just tried to listen to my body and let all the thoughts go on, or try to not force them away, just let them be there. And then I calmed down a lot. I was still pretty sad, but I wasn’t. after that I could still go out and continue.
(…)

Meditative practices were a core part of the curriculum, and several participants described that they experimented with the techniques in similar ways to this participant. The meditation exercises and techniques in themselves were described as an important source of inner peace and useful tools to establish more stability in their emotional life. Some participants described it as challenging for them to practice meditation in the class, especially the parts that involved attention toward the heart and breath. But even some of those who initially felt these exercises were challenging described these practices as quite powerful, sometimes to an extent that surprised them, especially when it came to their stabilizing potential. Helga described it this way:

And there was also a time, I thought I was going to be late so I was pretty stressed and had just managed to sit down, and I could feel my heart racing, and then we were supposed to start an exercise, and I could tell ‘Oh, now I am anxious, wow,’ and I got really stressed. And then we were supposed to feel our heartbeat. And then I could tell, okay, now it’s starting to help, now it’s beginning to…. When I started the exercise my heart was racing, and I thought it was really uncomfortable and I thought everybody would be able to see that it’s uncomfortable, and [I started to] imagine that someone is sitting with their eyes open and watching me. [However] When the exercise was over I was totally calm. So I think it had a really good effect on me.

Another participant who presented herself as a person unable to meditate because her ‘attention was all over the place’ experienced that the practices gave her more inner peace over time. So increased inner stability was an important topic when it came to what the participants described as the most important outcome of the course. It was also possible to see this as an implicit thematic pattern in the other themes the analysis brought up.

## Discussion

In analyzing the responses to the open-ended survey question and the interviews, we found that most of the answers revolved around finding new, less harsh ways of relating to oneself. This is in concordance with the findings from the quantitative part of the study, which showed a decrease in self-judgment and habitual negative self-directed thinking following the intervention (Dundas et al., 2017). In the present study, it became evident that all the informants we interviewed had experienced important changes in the way they related to themselves. Participants were more aware of how they treated themselves and managed to stay more supportive and friendlier toward themselves, particularly in the face of challenges. Even though awareness and self-support are presented as two separate subthemes in our results, these themes are clearly interrelated, as evident from the first quote from Maria (see p. 13). Maria describes how she now immediately notices when she starts to become highly self-critical, and is able to use this awareness as an opportunity to apply resources acquired from the course (such as attend to the breath) and be self-supporting in a difficult situation. While the students’ written responses to the open questions in the survey had pointed to the importance of becoming aware that one treated oneself too harshly, the interviews painted a more nuanced picture. There seemed to be a bi-directionality of awareness and self- support: while distress-awareness was necessary for responding supportively to inner distress, treating oneself kindly and with support seemed to open students up to greater awareness of distress and vulnerability.

Although the way awareness is facilitated differs between therapeutic approaches, awareness of thoughts and feelings as a premise of change resonates with thinking across different theoretical approaches, including cognitive therapy (Furlong and Oei, 2002), mindfulness-based cognitive therapy (Teasdale et al., 2000), and the tradition of mindfulness and self-compassion (Neff, 2003a; Frewen et al., 2008; Birnie et al., 2010). The interrelation between awareness and new actions reported by the participants in the present study points toward two interesting aspects of our findings: first, the participants’ stories showed the potential transformative power in increased awareness—but also how difficult this transformation of awareness into behavioral change can be. The participants described in various ways the paradoxical experience of finding the focus on being aware of distress and being kind to oneself familiar, almost simple, and at the same time really important in driving change, and difficult to live by. The psychoeducation and the practices during the course were experienced as important resources to make this transition from theoretical knowledge to actual experience and practical changes in everyday life. It seems that the repetition of exercises both in the meetings and between sessions facilitated an acceptance that practice is necessary. One participant even drew parallels to practicing first aid. This might carry significance, as being friendly toward oneself is easy as long as everything runs smoothly. It is when things becomes difficult that self-support becomes necessary (i.e., definition of self-compassion, Neff, 2003b).

Second, participants expressed surprise regarding how quickly things started to change once they adopted a slightly new focus. This was a very brief intervention—three meetings over the course of 2 weeks. Still, participants reported significant and meaningful psychological changes over this short period of time, an experiential result that we find validated through the RCT (Dundas et al., 2017) in this multi-method project. The participants’ stories show how they adopted the resources provided during the course and applied them as support in the context of their own life situations.

The findings imply that attending to distress over time might reduce distress. This is in contrast to the common belief that attending closely to distressing thoughts and emotions might increase the frequency of those very thoughts and emotions. Self-compassion training combines the awareness of distress with responses that might counteract the tendency for distress to escalate via rumination, shame, or other distressing emotions. The interviews indicated that participants had understood that being self-compassionate meant not adding insult to injury.

The interviews indicated that even a short intervention might be sufficient to facilitate positive change in non-clinical student samples, thus potentially serving a preventive effect for student mental health problems. Participants’ descriptions of new ways of relating to painful emotion resonate well with the increase in self-compassion, and decrease in anxiety and depression found in the quantitative data.

The way the participants experience increased agency and resilience is also in line with the findings from our quantitative study, which showed an increase in personal growth self-efficacy (Dundas et al., 2017). Several studies have shown that mindfulness training is associated with increased perceived agency and sense of control (e.g., Monshat, 2012; Moore and Martin, 2015). The present interviews show that not only mindfulness, but also self-compassion, may increase a feeling of agency and provide a useful tool when facing distressing situations and emotions.

Not all aspects of the course were experienced as positive to all participants. Difficulties with finding time to practice between sessions is a familiar problem for most people when learning a new skill, and students have a busy schedule with frequent lectures and deadlines associated with assignments, tests, and exams during the semester. The audio recorded guided meditations were, for this reason, kept short (15 min).

The finding that discussions between participants could be difficult for some might be explained in several ways. It could represent a cultural phenomenon, since ‘letting down one’s wall’ in dialog with a non-familiar peer is not as common in Scandinavia as in, for example, American culture. Also, conducting courses in a group of students who are unfamiliar with each other and the instructor, and who do not have enough time to get to know each other, may pose some challenges with regard to issues of perceived safety. In the current courses this challenge may have been exacerbated by some students knowing the instructor and being familiar with mindfulness and psychology in general, whilst others were not. On the other hand, other participants expressed satisfaction with the discussions and inquiry during the course, stating that this made them realize how common some of their own concerns were. As in any form of psychological interventions, the relationship between the instructor and the individual participants, as well as among the participants themselves, may be critical in deciding outcomes.

### Reflexivity, Scope, and Limitations

Reflexivity involves ‘an attitude of attending systematically to the context of knowledge construction, especially to the effect of the researcher, at every step of the research process’ (Malterud, 2001, p. 484). All the researchers were also clinicians with a relational, emotion-oriented, and experiential orientation to their clinical work, and most of them also to mindfulness based approaches. On the one hand, this probably sensitized the group to the exploration of issues related to emotional processes, self-self and self-other relations, and the therapeutic potentials of acceptance. On the other hand, it brings the possibility of a bias of overemphasizing emotional outcomes at the expense of cognitive aspects that are also documented to be of high importance in other studies (see for example Zessin et al., 2015). In the analysis, we have taken care to listen and to highlight positive experiences that also have to do with cognitive insights.

The first, second and fifth author also have training in self-compassion based approaches, and this also might be a source of bias. The dominant themes in the findings are easy to relate to theoretical approaches to self-compassion within psychology, e.g., Kristin Neff’s model. Neff’s concept of self-compassion is a central part of the curriculum of the course, and we hope that our findings reflect how the participants made use of this psychoeducational elements of the course, in addition to the practices that were taught. We invited the authors that were less invested in compassion-based approaches to take a critical role and examine elements of theoretical ‘impregnation’ in the analysis. However, in a qualitative study our reconceptualization of psychological phenomena will unavoidably play a role in the way we understand participants and their utterances, even when we are aware of our orientations—they also function as lenses. In our opinion, qualitative studies of outcome need to be supplemented by quantitative studies that offer stronger possibilities for disconfirmation of hypotheses.

The interview situation is a relationship between members of the academic staff and students from different fields of study, and the participants are also aware that the interviewers are mental health professionals. It is possible that conversations with peers could bring up other aspects, as happens in a focus group format. On the other hand, the individual interview format ensured confidentiality and opened up the possibility of in-depth exploration of the specific narratives of the participants. As found in the interviews, reflection and exploration together with peers can be experienced as challenging.

The themes selected for in-depth analysis in the 12 interviews are based upon written answers from the survey completed by the larger group of 94 participants. To a certain extent, this can secure representativeness of the themes that are chosen for further exploration. On the other hand, a content analysis based on templates achieved from analysis of a larger sample brings limitations to possible interpretations of the interviews, and exploration into experiences that are specific for the interviewed participants rather than those occurring frequently in the larger sample. Here, we chose to prioritize themes that were representative in the larger sample of participants.

There were limitations also connected to the intervention as such. Three sessions is a very short course. Many participants experienced the course as a little too short—they thought that one more session would have been useful. Participants experienced the assignment to listen to specific audio-recorded guided meditations between sessions as challenging. Many stated that it was hard to find time for them in a busy week schedule. Some of them also expressed feeling sleepy when they used them.

## Conclusion

A three-step qualitative analysis of participants experiences after a brief self-compassion course was conducted, consisting of a thematic analysis of finding from our online survey, content analysis of interviews with participants, and phenomenological exploration of these thematic content areas. The most central finding was that the participants experienced that they were more supportive and friendlier toward themselves when things were difficult or felt painful, having begun a process of treating themselves better in everyday life. As part of this process, they became more aware of how harshly they used to treat themselves in difficult situations. Many participants also described that their experience of painful emotions changed, both finding more relief in the fact that suffering is part of the human condition, and becoming more accepting to uncomfortable feelings. As a result of this, many participants also felt more stable and peaceful, and better able to cope with the everyday pressures and challenges in their student and personal lives.

## Data Availability Statement

The datasets generated for this study are available on request to the corresponding author.

## Ethics Statement

The studies involving human participants were reviewed and approved by the Regional Committee for Medical and Health Research Ethics – West. The patients/participants provided their written informed consent to participate in this study.

## Author Contributions

P-EB wrote the first draft of the manuscript. P-EB, ID, and SS contributed to the conception and design of the study. P-EB led the analysis of the data. P-EB, ID, AH, SS, and VW contributed to the analysis of the data. CM contributed with critical auditing of the analysis. P-EB, ID, SS, AH, VW, and CM wrote the sections of the manuscript. All authors contributed to the manuscript revision, and read and approved the submitted version.

## Conflict of Interest

The authors declare that the research was conducted in the absence of any commercial or financial relationships that could be construed as a potential conflict of interest.

## Appendix

### Interview Guide: Participants’ Experiences After a Self-Compassion Course.

First: read the participant’s code and today’s date

(1) What do you experience is the most important thing you got out of participating in the course?
(A) If the participant experiences that they treated themselves differently than before:
  - Can you tell me about a situation in which you noticed that you treated yourself in a different way than before?
If the participant does **not** describe a situation which consists of motivating themselves, you can ask:
  - Have you experienced situations in which you motivate yourself differently than before?
If the participant does **not** describe situations in which they failed, or were confronted with their own limitations:
  - Have you experienced situations in which you treat yourself differently when you fail, are unable to do something or somehow fall short?
  If the participant has **not** described a situation in which they treated themselves with kindness or care when they experienced painful/uncomfortable situations in relation to others, you can ask:
  - Have you experienced situations in which you noticed that you treat yourself in a different way than before when something has been painful or uncomfortable in relation to others?
  - Do you experience that participating in the course has affected your relationship to your performance^∗^ in situations in which your performance will be assessed? In which case, how so?
  - Do you experience that participating in the course has affected your relationship to other people in any way? In which case, how so?
(2) What was it that sparked your interest in participating in the course?
(3) What did you expect to get out of the course? How did your expectations compare to what you got out of the course?
(4) After you signed up for the course and before it started, did you start to read about self-compassion or work with the topic on your own?
(5) What was your experience of participating in the course sessions?
If the participant does not mention the small group discussions
  - What was your experience of talking to your neighbor (in the sessions)
If the participant does **not** mention the exercises
  - What was your experience of doing the exercises
(6) What was your experience of doing the homework?
(7) Was there anything in regards to the course that made you feel uncomfortable? Was there anything that you would describe as a downside to participating?
(8) What do you think about offering the course to all students who want to take it?
  Is there anything else that is important to you? Anything we forgot to ask? Anything you have not mentioned?
  ^∗^Word translated reflects, i.e., work, school type performance.
